# Metabolic and hormonal signatures in pre-manifest and manifest Huntington's disease patients

**DOI:** 10.3389/fphys.2014.00231

**Published:** 2014-06-23

**Authors:** Rui Wang, Christopher A. Ross, Huan Cai, Wei-Na Cong, Caitlin M. Daimon, Olga D. Carlson, Josephine M. Egan, Sana Siddiqui, Stuart Maudsley, Bronwen Martin

**Affiliations:** ^1^Metabolism Unit, National Institute on Aging, National Institutes of HealthBaltimore, MD, USA; ^2^Division of Neurobiology, Department of Psychiatry and Behavioral Sciences, Johns Hopkins University School of MedicineBaltimore, MD, USA; ^3^Departments of Neuroscience and Pharmacology and Molecular Sciences, Johns Hopkins University School of MedicineBaltimore, MD, USA; ^4^Diabetes Section, National Institute on Aging, National Institutes of HealthBaltimore, MD, USA; ^5^Receptor Pharmacology Unit, National Institute on Aging, National Institutes of HealthBaltimore, MD, USA; ^6^VIB Department of Molecular Genetics, University of AntwerpAntwerpen, Belgium

**Keywords:** Huntington's disease, pre-manifest, metabolic hormones, lipids, peripheral energy metabolism

## Abstract

Huntington's disease (HD) is an inherited neurodegenerative disorder typified by involuntary body movements, and psychiatric and cognitive abnormalities. Many HD patients also exhibit metabolic changes including progressive weight loss and appetite dysfunction. Here we have investigated metabolic function in pre-manifest and manifest HD subjects to establish an HD subject metabolic hormonal plasma signature. Individuals at risk for HD who have had predictive genetic testing showing the cytosine-adenine-guanine (CAG) expansion causative of HD, but who do not yet present signs and symptoms sufficient for the diagnosis of manifest HD are said to be “pre-manifest.” Pre-manifest and manifest HD patients, as well as both familial and non-familial controls, were evaluated for multiple peripheral metabolism signals including circulating levels of hormones, growth factors, lipids, and cytokines. Both pre-manifest and manifest HD subjects exhibited significantly reduced levels of circulating growth factors, including growth hormone and prolactin. HD-related changes in the levels of metabolic hormones such as ghrelin, glucagon, and amylin were also observed. Total cholesterol, HDL-C, and LDL-C were significantly decreased in HD subjects. C-reactive protein was significantly elevated in pre-manifest HD subjects. The observation of metabolic alterations, even in subjects considered to be in the pre-manifest stage of HD, suggests that in addition, and prior, to overt neuronal damage, HD affects metabolic hormone secretion and energy regulation, which may shed light on pathogenesis, and provide opportunities for biomarker development.

## Introduction

Huntington's disease (HD) is a neurodegenerative disorder involving the extrapyramidal motor system and is characterized by chorea, progressive dementia, and other psychiatric symptoms (Walker, 2007; Ross and Tabrizi, 2011; Weir et al., 2011). The incidence of HD is approximately 5–10 cases per 100,000 worldwide, making it one of the most common inherited neurodegenerative disorders. HD is caused by a dominant genetic mutation in the *huntingtin* (*HTT*) gene that results in an expanded tri-nucleotide repeat of cytosine-adenine-guanine (CAG). This CAG repeat codes for an expanded polyglutamine repeat near the N-terminus of the HTT protein, which undergoes a conformational change, and causes cellular damage (Walker, 2007). The areas of the brain most affected by mutant HTT are the striatum, followed by the cerebral cortex, and then other brain regions. The onset of HD typically occurs around the age of 30–40, and as the disease develops patients progressively lose independence and, eventually, die.

Many HD patients suffer from weight loss and as a result it is believed that metabolic dysfunction contributes to HD pathogenesis (Martin et al., 2008; Van Der Burg et al., 2009; Cai et al., 2012). Aziz et al. (2010a) reported that in HD patients, energy expenditure in a fasted state is increased compared to healthy control subjects, suggesting that a hypermetabolic state could contribute to the observed increase in energy expenditure in HD (Aziz et al., 2010a). Further reinforcing the presence of somatic metabolic dysfunction in HD, abnormal insulin, and leptin secretion rates have been shown to be positively correlated with higher CAG repeat number (Aziz et al., 2010b), which may contribute to the weight loss that is evident in many HD patients. The R6/2 transgenic mouse, which express exon 1 of human HD gene with around 150 CAG repeats (Mangiarini et al., 1996), is the most widely used mouse model to study HD pathology. Blood glucose levels in R6/2 HD mice are significantly higher than in wild type controls. Reduced insulin production is also evident in this model (Andreassen et al., 2002). In addition, the N171-82Q murine HD model expressing a 171 amino acid- N-terminal human HTT cDNA insertion giving rise to an 82 CAG repeat expansion, displays multiple aspects of diabetic-like pathophysiology (Martin et al., 2009, 2012). It has been demonstrated that anti-diabetic therapeutics can ameliorate the metabolic dysfunction present in this model (Martin et al., 2012). Deletion of huntingtin-associated protein 1 (HAP1), in pancreatic beta cells has also been shown to result in impaired glucose tolerance by reducing glucose-mediated insulin release in these mice (Kaushik et al., 2011).

In the current study, we recruited both pre-manifest and manifest HD subjects in order to conduct an investigation of the metabolic profile in HD, through the measurement of brain and metabolic hormone levels; lipid profiling and cytokine levels. We recruited familial control groups, to primarily control for lifestyle variables, and Baltimore Longitudinal Study of Aging (BLSA) control groups to effectively control for age, gender composition and BMI influences. We found that growth hormone (GH) and prolactin were significantly decreased in the manifest HD subjects. In the fasted state, HD subjects presented similar blood sugar, insulin, and adiponectin levels compared to all control groups, whereas plasma ghrelin, glucagon, and amylin levels were significantly altered. Total cholesterol (CH) levels as well as the high-density lipoprotein-cholesterol (HDL-C) and low-density lipoprotein-cholesterol (LDL-C) levels in HD subjects were decreased. Our findings indicate that HD patients possess impaired energy homeostasis and abnormal hormone levels. This state can be detected as distinctive metabolic plasma “signature,” even in the early pre-manifest stage of HD. These findings suggest that, in addition to neuronal damage, HD pathogenesis could involve widespread metabolic dysfunction.

## Materials and methods

### Research subjects

Institutional Review Board approval was obtained from the National Institute on Aging, and informed written consent was obtained from all participants. Fifteen pre-manifest and eight manifest HD subjects, as well as 16 control subjects from the patient's families were recruited for this study (Johns Hopkins familial cohort). Due to the difficulty of matching for age, body mass index (BMI), and gender in familial controls, we employed additional control subjects from the BLSA. The BLSA subjects are healthy people without diagnosed metabolic or neuronal diseases. With the BLSA control subjects we were able to avoid confounding factors such as body weight, age, and gender. Thus, BLSA control groups adequately control for age, gender, and BMI, while the Johns Hopkins familial control group control for the socioeconomic status and prevalent stress levels in the HD subject groups, as familial caregivers experience similar stressors/home environment as the HD subjects. Hence the BLSA pre-manifest control group matched pre-manifest subjects and the BLSA manifest control group matched the manifest subjects. Characteristics of all the subject groups are listed in Table 1. Blood samples were collected separately during each subject's visit to the hospital. Blood samples of subjects from Johns Hopkins hospital were taken in the morning after an over-night fast. Plasma samples were centrifuged at 3000 rpm at 4°C and were subsequently stored at −80°C until processed. BLSA plasma samples were taken in the morning after an over-night fast and prepared using the same methodology, and as described previously (Driscoll et al., 2012). The homeostatic model assessment of insulin resistance (HOMA-IR) was calculated as follows: insulin (mU/L) × glucose (mg/dL)/405 (Matthews et al., 1985). BMI was calculated as: body weight (kg)/height (m^2^) according to previously established standards (Golden et al., 2010).

**Table 1:** **Subject characteristics**.

**Subjects**	**Age**	**Gender (number of male/female)**	**Body mass index**	**Number of CAG repeats**
Familial control	48.2 ± 1.9	8/8	26.2 ± 1.4	20.6 ± 0.14
BLSA pre-manifest control	46 ± 2.0	10/5	26.5 ± 1.0	−
BLSA manifest control	57 ± 3.7	5/3	25.4 ± 1.2	−
Pre-manifest subjects	46.8 ± 2.1	10/5	27.4 ± 1.5	42.3 ± 0.09
Manifest subjects	57.6 ± 4.1^*^	5/3	24.9 ± 1.4	42.5 ± 0.12

* p ≤ 0.01, Significant difference was observed when compared with the familial control group.

Values are mean ± s.e.m. for age, body mass index, and number of CAG repeats.

### Plasma hormone measurements

Insulin, leptin, GIP (Gastric inhibitory peptide), amylin, PYY (Peptide YY), and PP (Pancreatic polypeptide) were assayed using a human gut hormone multiplex kit according to the manufacturer's instructions (Millipore, Billerica, MA). Intra-assay variation was lower than 11%. Adiponectin levels were measured by radioimmunoassay (Millipore, Billerica, MA) and the intra-assay variation for this was from 6.90 to 9.25%. Total plasma ghrelin levels were measured using an ELISA assay kit from Millipore according to the manufacturer's instructions. Glucagon levels were assayed using a Millipore RIA kit. Brain-derived neurotrophic factor (BDNF), GH, Agouti-related protein (AgRP), and prolactin were measured with a Human Brain-Derived/Pituitary Protein Multiplex Panel assay kit according to the manufacturer's instructions (Millipore): intra-assay variation was lower than 10%. Cytokines were assayed using a human cytokine/chemokine assay kit according to the manufacturer's instructions (Millipore): intra-assay variation was lower than 10.5%. C-reactive protein (CRP) was measured using an ALPCO ELISA kit according to the manufacturer's instructions (ALPCO Diagnostics, Salem NH), and the intra-assay variation was 5.5–6.0%.

### Glucose and lipid measurements

Plasma glucose levels were measured using a glucose assay kit (Cayman Chemical Company, Ann Arbor, MI). CH, HDL-C, LDL-C, triacylglycerols, and free fatty acid levels were measured using enzymatic assay kits according to the manufacturer's instructions (Wako Pure Chemical Industries, Ltd. Japan).

### Statistical analyses

One-Way ANOVA was used for the statistical analysis, and Bonferroni's multiple-comparison test was used for specific comparisons within the five groups using the R software package. Probability (*p*) values of < 0.05 were considered statistically significant for one-to-one comparisons. We further performed linear regression analysis of hormones levels of individual subjects by age or BMI, respectively. Standard linear regression analyses were performed using GraphPad Prism v5.0. Probability (*p*) values < 0.05 were considered to have a slope statistically different from zero.

## Results

### Altered growth factors in HD subject plasma

**Figure 1:**
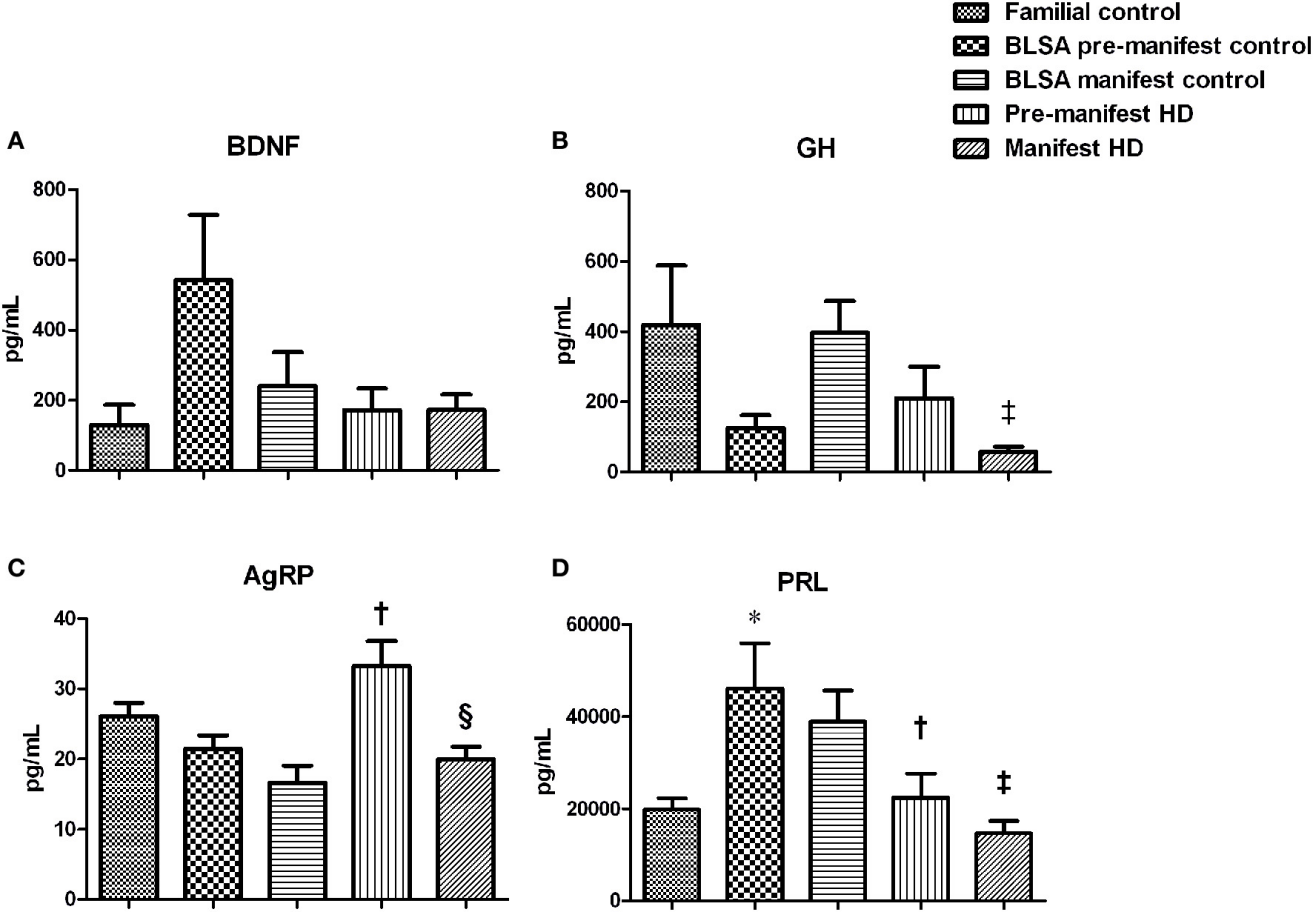
**Growth hormone changes in HD subjects**. Plasma BDNF, growth hormone (GH), Agouti-related protein (AgRP), and prolactin (PRL) were measured using the Millipore brain-derived protein panel. Data are mean ± s.e.m., *n* = 8–16. Statistical analysis was performed using One-Way ANOVA followed by Bonferroni's multiple-comparison test, *p* ≤ 0.05 (^*^, †, ‡, §) and *p* ≤ 0.01 (^**^, ††, ‡‡, §§) were considered statistically significant. ^*^ Represents significant difference against familial control; ^†^ represents significant difference against BLSA pre-manifest control; ^‡^ represents significant difference against BLSA manifest control and ^§^ represent significant difference against pre-manifest HD subjects. No change was found in BDNF levels **(A)**. Reduction in GH was observed in manifest HD subjects **(B)**. Elevation of AgRP was found in pre-manifest group **(C)**. Reduction of PRL was observed in both pre- and manifest subjects **(D)**.

As HD pathophysiology is tightly linked to neurodegenerative processes, and potentially peripheral metabolic dysfunction, we assessed the circulating levels of multiple factors associated with both of these functions (Figure 1). No difference in BDNF levels was observed among all five groups (Figure 1A). Compared to the BLSA control group (396.97 ± 84.67 pg/mL), manifest HD subjects showed significantly decreased levels (57.94 ± 14.28 pg/mL) of circulating GH (Figure 1B, *p* = 0.001). A similar, non-significant, trend for manifest HD reduced GH (57.94 ± 14. 28 pg/mL), was also observed compared to the familial control group (419.98 ± 169.96 pg/mL, *p* = 0.08). No difference was observed in the pre-manifest group. Compared to the BLSA control group (21.48 ± 1.18 pg/mL), pre-manifest HD subjects showed a significant increase in circulating AgRP (33.28 ± 3.05 pg/mL, *p* = 0.007) (Figure 1C), and the same trend was also observed when compared to the familial controls (25.14 ± 1.60 pg/mL, *p* = 0.056). This distinction for AgRP was not repeated in the manifest HD subjects. Between the two HD groups, we also observed that there was a reduction in manifest HD AgRP levels when compared with the pre-manifest HD. With respect to PRL levels we found a consistent effect, i.e., reduced PRL levels, in both pre-manifest and manifest HD subjects when compared to the respective BLSA control groups (Figure 1D). However, this effect was not observed when compared with the familial control group.

### Metabolic hormone changes in HD subject plasma

**Figure 2:**
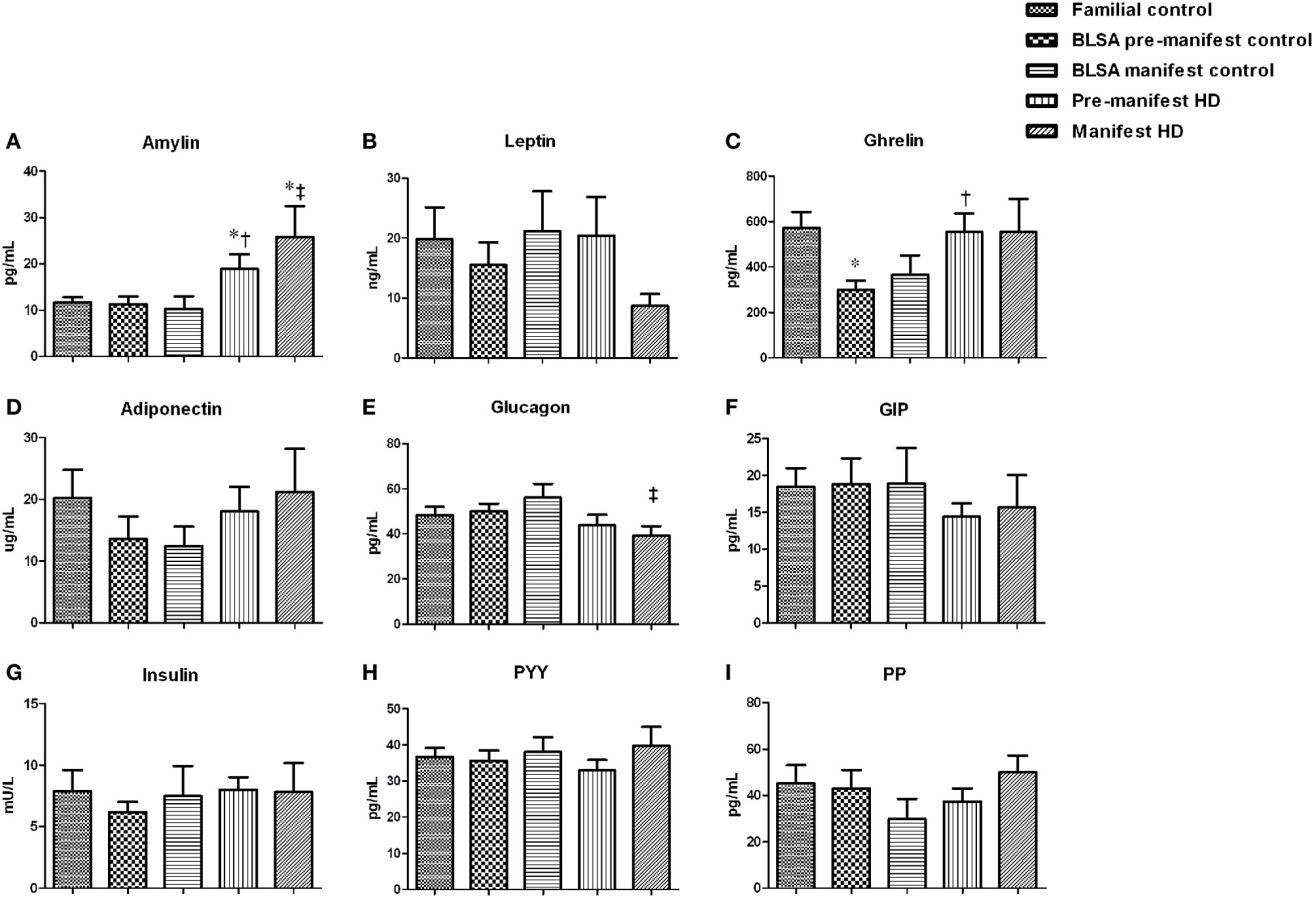
**Metabolic hormone levels in HD subjects**. Plasma levels of amylin, leptin, ghrelin, adiponectin, glucagon, Gastric inhibitory peptide (GIP), insulin, peptide YY (PYY), and pancreatic polypeptide (PP) were measured. Data are means ± s.e.m., *n* = 8–16. Statistical analysis was performed using One-Way ANOVA followed by Bonferroni's multiple-comparison test, *p* ≤ 0.05 (^*^, †, ‡, §) and *p* ≤ 0.01 (^**^, ††, ‡‡, §§) were considered statistically significant. ^*^ Represents significant difference against familial control; ^†^ represents significant difference against BLSA pre-manifest control; ^‡^ represents significant difference against BLSA manifest control and ^§^ represent significant difference against pre-manifest HD subjects. Amylin levels increased in both pre- and manifest subjects **(A)**. HD manifest subjects have a trend of reduced leptin levels **(B)**. Both pre- and manifest subjects have increased ghrelin **(C)** with a significant difference in the pre-manifest group. No significant changes were observed for adiponectin **(D)**. Glucagon was significantly reduced in the manifest subjects **(E)**. No significant differences were found in the levels of GIP **(F)**, insulin **(G)**, PYY **(H)**, and PP **(I)**.

To further investigate the potential metabolic aspects of HD in pre-manifest and manifest subjects, we measured the circulating concentrations of a series of energy-related hormones including amylin, leptin, ghrelin, adiponectin, glucagon, GIP, insulin, PYY, and PP in fasted-state plasma (Figure 2). Amylin levels were found to be significantly elevated in both pre-manifest and manifest HD subjects (Figure 2A). Leptin levels were not different in pre-manifest HD subjects, however in the manifest subjects there was a trend for decreased leptin levels (Figure 2B, *p* = 0.13 for familial controls and *p* = 0.06 for BLSA controls). Compared with the BLSA control group (298.52 ± 38.26 pg/mL), a significant increase in ghrelin levels in pre-manifest HD subjects (554.26 ± 79.48 pg/mL) was observed (Figure 2C, *p* = 0.009). Adiponectin levels were comparable among all five groups (Figure 2D). We observed, only in the manifest HD subjects (39.21 ± 4.03 pg/mL) compared to the BLSA control (56.02 ± 5.75 pg/mL), a significant reduction in circulating glucagon levels (Figure 2E, *p* = 0.045). GIP, insulin, PYY, and PP levels were not different among all five groups (Figures 2F–I). In addition, we assessed fasting glucose levels: as with insulin levels, we found no difference between any groups.

We also performed linear regression analysis using hormones and factors against age and BMI. With respect to HD-associated significant alterations in these regression relationships we found that leptin- and adiponectin-BMI interactions were affected. Leptin levels positively correlated with BMI in all three control groups (Figures S1A–C). Whereas in both pre-manifest HD and manifest HD subjects, no such correlation was observed (Figures S1D,E). Furthermore, we found in all control groups that the adiponectin levels were negatively correlated with BMI (Figures S2A–C), while in pre-manifest HD and manifest HD groups, no correlation was found (Figures S2D,E).

### Plasma lipid profile in pre-manifest and manifest HD subjects

**Figure 3:**
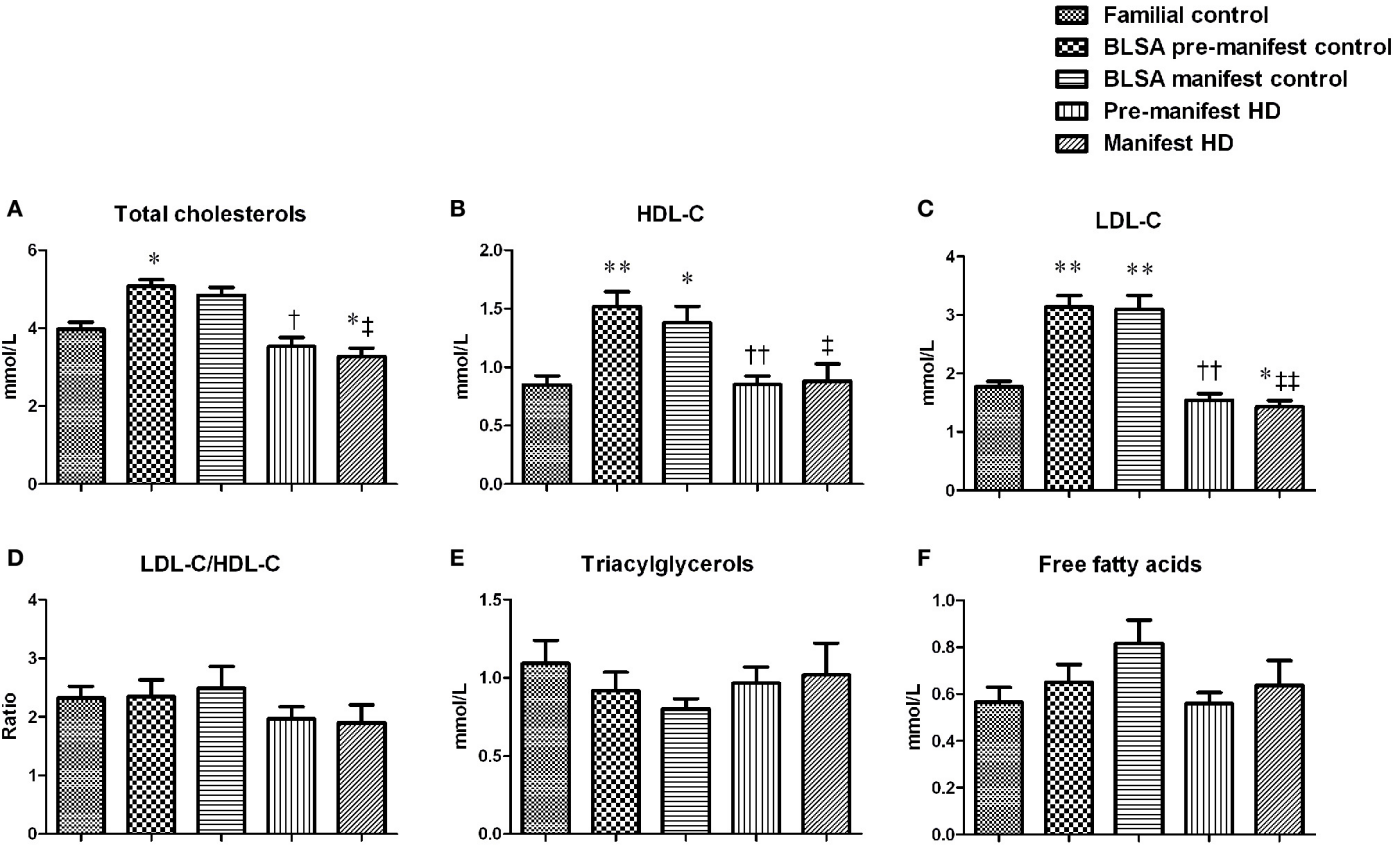
**HD subject plasma lipid profiles**. Lipids were measured using enzymatic methods. Data are means ± s.e.m., *n* = 8–16. Statistical analysis was performed using One-Way ANOVA followed by Bonferroni's multiple-comparison test, *p* ≤ 0.05 (^*^, †, ‡, §) and *p* ≤ 0.01 (^**^, ††, ‡‡, §§) were considered statistically significant. ^*^ Represents significant difference against familial control; ^†^ represents significant difference against BLSA pre-manifest control; ^‡^ represents significant difference against BLSA manifest control and ^§^ represent significant difference against pre-manifest HD subjects. Both pre- and manifest HD subjects have reduced cholesterol, HDL and LDL levels **(A–C)** while no difference was found in the ratio of LDL/HDL **(D)**, nor the levels of triacylglycerols **(E)** and free fatty acids **(F)**.

We next assessed the circulating lipid profiles of HD subjects to investigate how their metabolic status may affect lipid metabolism (Figure 3). Both pre-manifest and manifest HD subjects demonstrated significantly decreased CH levels compared to the BLSA control groups (Figure 3A). This significance was also seen in the manifest HD group when compared with the familial controls. With respect to the HDL-C (Figure 3B), the levels among the HD and familial control groups were comparable. Whereas decreased levels were observed in the pre- and manifest HD groups, compared with their BLSA control groups, LDL-C levels of pre-manifest HD were decreased relative to the BLSA pre-manifest controls. LDL-C levels of manifest HD subjects were less than both familial and BLSA control groups (Figure 3C). No differences in the LDL-C/HDL-C ratios were observed for pre-manifest or manifest HD subjects (Figure 3D). No differences in triacylglycerols (Figure 3E) or free fatty acids levels (Figure 3F) were observed.

### Circulating inflammatory factors in HD subjects

**Figure 4:**
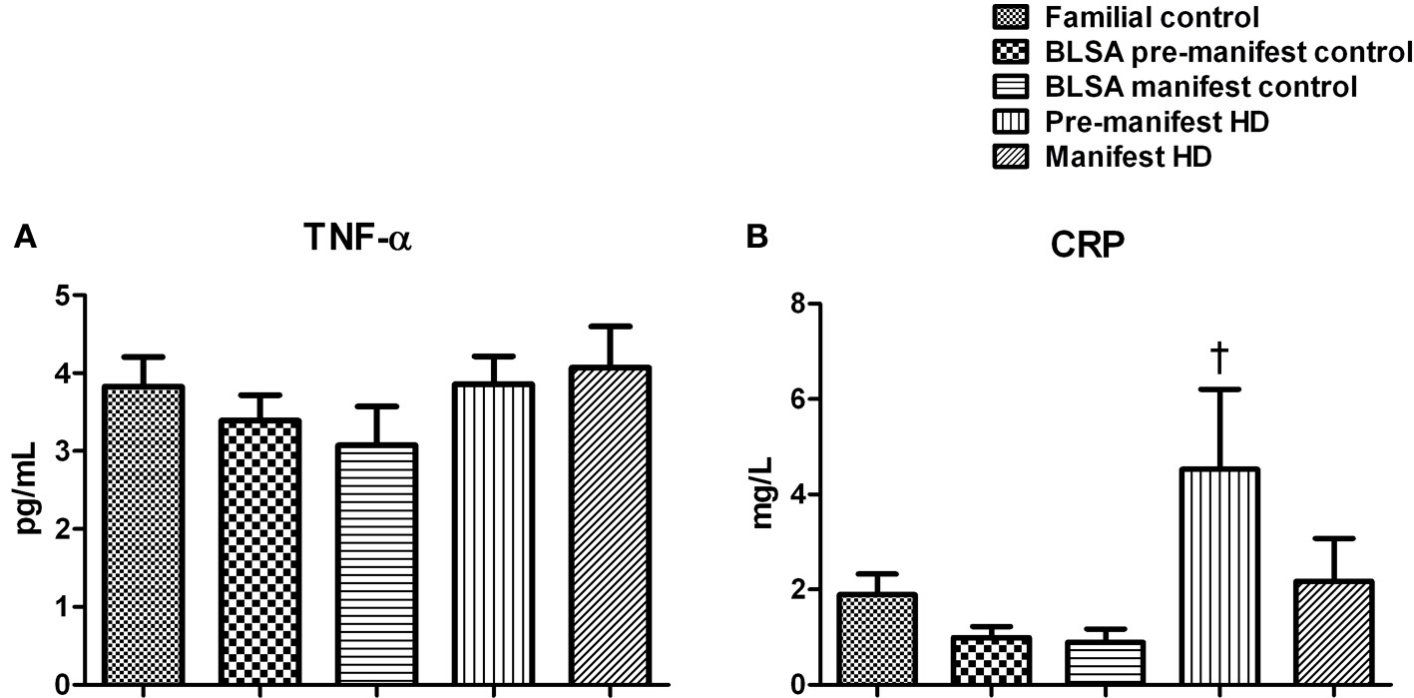
**Plasma levels of TNF-α and C-reactive protein in HD**. Plasma levels of TNF-α and C-reactive protein (CRP) were measured. Data are means ± s.e.m., *n* = 8–16. Statistical analysis was performed using One-Way ANOVA followed by Bonferroni's multiple-comparison test, *p* ≤0.05 (^*^, †, ‡, §) and *p* ≤ 0.01 (^**^, ††, ‡‡, §§) were considered statistically significant. ^*^ Represents significant difference against familial control; ^†^ represents significant difference against BLSA pre-manifest control; ^‡^ represents significant difference against BLSA manifest control; and ^§^ represent significant difference against pre-manifest HD subjects. No difference was observed in the levels of TNF-α. There was a non-significant trend for increase in TNF-α in the manifest HD group. CRP was increased in the pre-manifest HD subject plasma compared to BLSA pre-manifest controls.

We have previously demonstrated a strong association between neurodegeneration, metabolic dysfunction, and circulating inflammatory factors (Johnson et al., 2007; Chadwick et al., 2008; Stranahan et al., 2012), therefore we assessed circulating inflammatory mediators including tumor necrosis factor alpha (TNF-α), CRP as well as interleukins 1, 6, and 10 (IL-1, IL-6, IL-10) in pre-manifest and manifest HD subjects. As shown in Figure 4, we found no differences in circulating TNF-α between any of the experimental groups (Figure 4A). With respect to CRP levels we found a significant elevation in CRP levels in pre-manifest HD subjects compared to the BLSA pre-manifest control group (Figure 4B). A similar trend was also observed when compared with the familial controls. However, this elevation was not evident in the manifest HD group. Circulating levels of the interleukins IL-6, IL-1α, IL-1β ± and IL-10 were mostly below 2–3 pg/mL, such low and minimally-detectable levels are typical of human subjects that are not presenting excessive immune responses (Yamamura et al., 1998; Licastro et al., 2000).

### HD subject plasma signature

**Figure 5:**
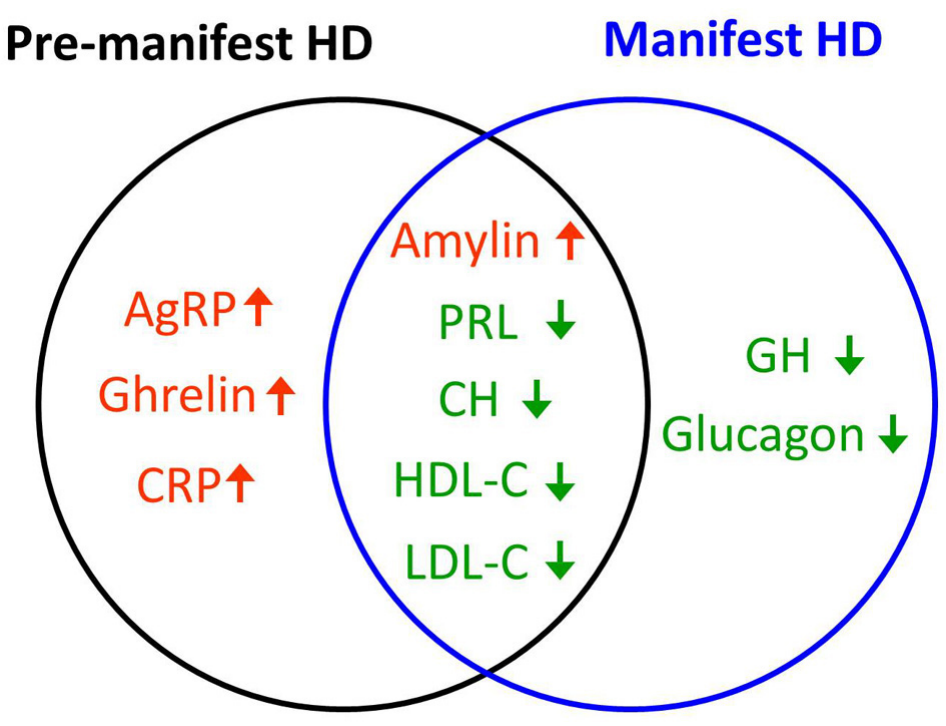
**HD plasma signature**. Hormones and cytokines that are uniquely up- (red) or down-regulated (green) in pre-manifest (black circle) or manifest subjects (blue circle) as well as those that are commonly up- or down-regulated in both pre- and manifest HD groups are shown in the Venn diagram. Amylin is the only factor that was commonly up-regulated in both pre- and manifest HD group. Prolactin (PRL), total cholesterol (CH), HDL-C, and LDL-C were commonly down-regulated in both HD groups. Factors that were uniquely up-regulated in the pre-manifest HD group were AgRP, ghrelin, and C-reactive protein (CRP). Factors that were uniquely down-regulated in the manifest HD group were growth hormone (GH) and glucagon.

Based on the profiles we derived of plasma hormones, cytokines and lipids, we have generated a preliminary HD subject plasma signature, i.e., a series of factors the expression of which is indicative of the pre-manifest and manifest states in HD (Figure 5). Factors that were uniquely changed (up-regulated) in pre-manifest group were AgRP, ghrelin, and CRP while in the manifest HD group factors that were uniquely changed (down-regulated) were GH and glucagon. Factors that were commonly changed in both pre- and manifest groups were amylin, prolactin, CH, HDL-C, and LDL-C, among which amylin is the only factor that was elevated. The remaining four were decreased in both pre- and manifest HD groups.

## Discussion

In this study we investigated the alterations of circulating metabolic factors in HD subjects. We found that amylin levels were significantly increased in both pre-manifest and manifest HD subjects. GH and Glucagon levels were decreased in the manifest HD subjects compared to BLSA controls. We also found that levels of AgRP, Ghrelin, and CRP were increased in pre-manifest HD, PRL levels were reduced in both pre-manifest and manifest HD cases compared to the BLSA control group. CH as well as HDL-C and LDL-C were decreased in both HD subject groups. Pre-manifest HD subjects presented increased CRP levels. The correlation between leptin levels and BMI was diminished in both pre- and manifest HD groups, and the negative correlation between adiponectin and BMI was diminished in both pre and manifest HD groups.

Amylin is co-secreted with insulin from pancreatic islets in response to food ingestion (Butler et al., 1990; Pittner et al., 1994). Amylin is anti-orexigenic and suppresses gastric motility and glucose uptake (Macdonald, 1997; Gedulin et al., 2006; Potes et al., 2012). It is also known as islet amyloid peptide and is closely related to the beta-amyloid peptide that is associated with the cytotoxic and neurodegenerative aspects of Alzheimer's disease (Moreno-Gonzalez and Soto, 2011; Burke et al., 2013). Elevated fasting amylin levels observed in the current study raised an interesting speculation that in HD subjects (even in the pre-manifest subjects) abnormal control of gastrointestinal function may contribute to the metabolic aspect of the pathology in this disease. Amylin, like other amyloid molecules, often undergoes conformational changes to form ion channel-like structures that may destabilize cellular ionic homeostasis and thus induce cellular degeneration (Quist et al., 2005). Elevated amylin levels are typically associated with dysglycemia and pancreatic beta-cell dysfunction in Type 2 diabetes (Koda et al., 1992; Lorenzo et al., 1994; Ye et al., 2001). It is therefore interesting, and potentially functionally-relevant, to speculate that the regulation of amylin levels could be a critical molecular connection between diverse forms of neurodegenerative diseases that also possess a metabolic deficit (Martin et al., 2008; Cai et al., 2012). With our identification of amylin as a potential therapeutic target of the peripheral metabolic dysfunction in HD, it is possible that pharmacological manipulation of such a factor could represent an important avenue for future anti-neurodegenerative therapies (Martin et al., 2005; Chapter et al., 2010). With respect to the relationship between HD pathology and somatic metabolism it is interesting to note that both amylin and ghrelin functionally target the gut (Butler et al., 1990). Our results therefore demonstrate an interesting phenomenon of a simultaneous increase of both an appetite stimulator, ghrelin, and an appetite suppressor, amylin. This suggests that HD patients possess multiple abnormalities in the hormone balance for the regulation of food intake and absorption. It has been demonstrated that in HD patients, energy expenditure increases with disease duration, but not with a greater degree of motor or functional impairment (Aziz et al., 2010a). Taken together these findings suggest that HD pathology and metabolic dysfunction may be initiated as a nutrient uptake and energy utilization deficiency, which then result in weight loss and dysglycemia.

Our observation of the relatively normal fasting glucose and insulin levels, as well as normal range HOMA-IR values in our subjects suggests that neither pre- or manifest HD subjects have impaired glucose tolerance or insulin resistance. Previous studies reported that there was no difference in glucose tolerance or insulin released during oral glucose tolerance test (OGTT) in early or middle stage HD patients (Kremer et al., 1989; Boesgaard et al., 2009). However, other studies, including those employing OGTTs, have demonstrated correlations between abnormal glycemic control and HD pathophysiology in patients (Podolsky et al., 1972; Podolsky and Leopold, 1977). These studies together with our findings suggest that the etiology of HD may be associated more with general impairment of hormone release and that glucose regulatory issues caused by this disruption might exist as an indirect effect.

Glucagon levels were decreased in the manifest HD subjects compared with BLSA manifest controls and the same trend was seen when compared with the familial controls. Our finding of reductions in plasma glucagon level is in accordance with previous studies in R6/2 transgenic mice (Hurlbert et al., 1999). In addition to its crucial role in maintaining effective energy metabolism glucagon can also act as a neuroprotective agent by reducing the neurotoxic glutamate (Fanne et al., 2011a,b), therefore reduction of this metabolic regulator may suggest an important role in mediating HD pathogenesis.

Leptin levels in plasma are usually acutely regulated by fasting and refeeding, and are also highly correlated to body fat mass in humans (Maffei et al., 1995; Kolaczynski et al., 1996). In our study we found a trend for reduced leptin levels in manifest HD subjects. As with the connection between HD pathophysiology and glycemic control, the specific relationship between leptin and HD appears highly nuanced. For example, a recent study has demonstrated that while circulating levels of leptin are similar between control and HD patients there is an increase in the rate of leptin secretion of HD patients (Aziz et al., 2010b). Another study has indicated that leptin levels can be doubled in pre-manifest HD patients compared to control, albeit in a non-significant manner (Goodman and Barker, 2011). Indicative of the complexity of energy balance networks, significant reductions in circulating leptin levels has also been reported (Popovic et al., 2004). In accordance with previous reports, our linear regression test showed that in both pre- and manifest HD groups the correlation between leptin and BMI was lost, also suggesting abnormal leptin secretion in the HD subjects. It should be noted that with respect to the disruption of leptin-secretion in HD, the small sample size and mixed gender may contribute to the degree of variability observed. With improvement in sample size and gender difference it is likely that some clinical consensus can be achieved to the important hormonal systems.

Adiponectin is secreted from adipose tissue and reported to be negatively correlated with body fat mass (Gavrila et al., 2003). We found that the adiponectin levels in all the three control groups showed a significant negative correlation with BMI. However, this correlation was attenuated in both pre- and manifest HD subjects. Adiponectin mediates insulin sensitization in adipose tissue via support of insulin signaling and kinase pathways (Ballantyne et al., 2005). Reductions in adiponectin levels have also been associated with obesity and insulin resistance (Kadowaki et al., 2006). In fact, in two HD animal models, reductions in adiponectin levels occur before body weight loss, suggesting that disruptions in adipocytokine secretion may be intrinsic to HD pathology (Phan et al., 2009). Together with our findings, the consistent changes of leptin and adiponectin in HD, suggests abnormal adipocyte function is linked to the metabolic alterations in HD pathophysiology.

In addition to the multiple altered appetite-associated hormones, we found that both GH and prolactin levels were decreased in manifest HD subjects. Not only is the signaling activity of prolactin associated with nervous system protective behavior (Torner et al., 2009), prolactin is also linked with diabetic pathology, metabolic syndrome and inflammatory conditions (Balbach et al., 2013; Chirico et al., 2013). Impaired prolactin responses have been reported in the HD patients (Hayden et al., 1977) as a result of abnormal dopaminergic activity in hypothalamus. Our results suggest that changes of basal levels of prolactin are detectable early in pre-manifest HD subjects. GH actions often synergize with those of prolactin as they are both situated in the hypothalamic-pituitary system that links both central and peripheral control of energy metabolism and cellular growth. In the periphery both prolactin and GH can interact to affect energy metabolism (Schäffler et al., 2005) and immune/inflammatory functions (Redelman et al., 2008). Reduced GH and prolactin levels suggest that the HD patients may possess a dysfunctional hypothalamic-pituitary system (Phelps, 1994; Phelps and Hurley, 1999), which potentially affects food intake and energy balance, leading to further weight loss and energy insufficiency (Gerardo-Gettens et al., 1989; Auffret et al., 2012).

We observed significantly decreased levels of cholesterol, HDL-C and LDL-C in both pre- and manifest HD subjects. For manifest HD subjects, the same reductions in CH and LDL-C were seen when compared with both familial and BLSA controls, in accordance with previous reports (Markianos et al., 2008). In addition to impaired cholesterol synthesis in HD patients (Valenza et al., 2005), cholesterol changes may also be the result of dysfunction related to food digestion and absorption, which correlates with our observation of elevated amylin levels. Cholesterol is important for adult brain neuron membrane formation, myelination and synaptic plasticity (Mauch et al., 2001; Hering et al., 2003; Quan et al., 2003). Studies have also demonstrated that circulating cholesterol concentration is related to dementia and emotion changes (Evans et al., 2000; Dietschy and Turley, 2004). Impaired cholesterol metabolism was also reported in HD patients recently (Leoni et al., 2011). In their study reduced plasma level of 24S-hydroxycholesterol (24OHC), the brain specific elimination product of cholesterol, and reduced levels of the cholesterol precursors lanosterol and lathosterol were observed, suggesting a critical role of cholesterol in HD pathology (Leoni et al., 2011).

We found that CRP was significantly elevated in the pre-manifest HD patients (Figure 4B), as has also been reported by Stoy et al. (2005). A CRP level of this nature is indicative of the inflammation of coronary vessels and is related to atherosclerotic process (Lagrand et al., 1999). It is also interesting to note that in a murine HD model the presence of a progressive cardiac dysfunction associated with impaired myocardial contractility, reduced left ventricular pressure and a significantly reduced coronary blood flow has been demonstrated (Wood et al., 2012). Therefore, it is possible that elevated CRP levels in HD could be strongly associated with potential cardiac and cardiovascular dysfunction, which may further exacerbate the disorder. As we have shown that amylin is elevated in HD patients, this may also cause cardiovascular issues as amylin is linked to the generation of stenosing deposits or plaques in the thoracic aorta (Westermark and Westermark, 2011). It is therefore likely that these two factors, amylin and CRP, could interact to disrupt both metabolic and cardiovascular function in HD. Therefore, pharmacotherapeutic targeting of both or either of these circulating hormone systems in patients could present an important new avenue for remedial research.

In conclusion, our study indicates that abnormal hormone levels in HD patients may contribute to the progression of energy imbalance. The hormones altered in HD affect not only food intake and absorption, potentially causing weight loss and malnutrition, but also impair interactions among metabolic systems, thereby inducing more global energy deficits that, ultimately, could adversely impact CNS function. Our study on both pre- and manifest HD subjects suggests that metabolic dysfunction occurs before the onset of diagnosable HD symptomology. It is possible that along with its deleterious actions on central nervous tissue, aggregated mutant HTT protein may also have direct effects on peripheral tissues such as the intestine, pancreas and adipose tissue. We have previously demonstrated in animal models of HD that therapeutic targeting of these peripheral energy-regulating organs can be beneficial for HD pathologies (Martin et al., 2012). Therefore, our current study further supports the idea that a combined therapeutic strategy, targeting both central and peripheral systems, may prove effective at ameliorating this devastating genetic disorder.

## Supporting grants

AG000915-01, AG000917-01, NINDS 16375, HDSA COE grant.

### Conflict of interest statement

The authors declare that the research was conducted in the absence of any commercial or financial relationships that could be construed as a potential conflict of interest.

## Supplementary material

Figure S1 **Correlation between leptin levels and the body mass index (BMI)**. Linear regression test was performed using leptin levels against the BMI. *p* ≤ 0.05 was considered statistically significant with respect to the linear correlations' deviation from a zero slope. r^2^ correlation was calculated by GraphPad Prism v5.0. In familial, BLSA pre-manifest, and manifest control groups, leptin levels were positively correlated with the BMI **(A–C)**; in pre-manifest HD and manifest HD groups no correlation was observed **(D,E)**.

Figure S2 **Correlation between adiponectin levels and the body mass index (BMI)**. Linear regression test was performed using adiponectin levels against the BMI. *p* ≤ 0.05 was considered statistically significant with respect to the linear correlations' deviation from a zero slope. r^2^ correlation was calculated by GraphPad Prism v5.0. In familial, BLSA pre-manifest, and manifest control groups, adiponectin levels were negatively correlated with the BMI **(A–C)**; whereas in pre-manifest HD and manifest HD groups no such correlation was observed **(D,E)**.
