# Supplementary figures and images for: Metabolic and hormonal signatures in pre-manifest and manifest Huntington's disease patients
Source: Front Physiol. 2014 Jun 23;5:231. doi: 10.3389/fphys.2014.00231 (PMC4066441; doi:10.3389/fphys.2014.00231)

**A**

Leptin

 $p=0.020$   
 $r^2=0.348$ 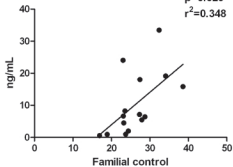**B**

Leptin

 $p=0.015$   
 $r^2=0.376$ 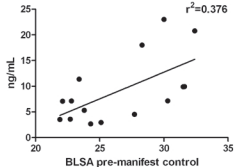**C**

Leptin

 $p=0.046$   
 $r^2=0.510$ 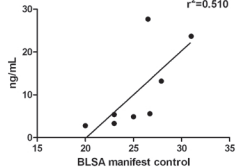**D**

Leptin

 $p=0.140$   
 $r^2=0.159$ 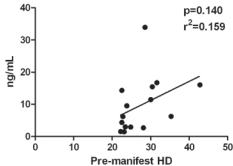**E**

Leptin

 $p=0.191$   
 $r^2=0.265$ 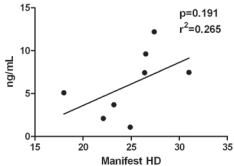

Supplement: Figure S1 — Correlation between leptin levels and the body mass index (BMI). Linear regression test was performed using leptin levels against the BMI. p ≤ 0.05 was considered statistically significant with respect to the linear correlations' deviation from a zero slope. r2 correlation was calculated by GraphPad Prism v5.0. In familial, BLSA pre-manifest, and manifest control groups, leptin levels were positively correlated with the BMI (A–C); in pre-manifest HD and manifest HD groups no correlation was observed (D,E). [file Presentation1.PDF]

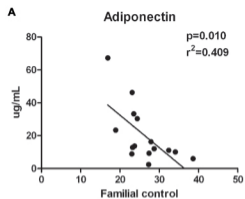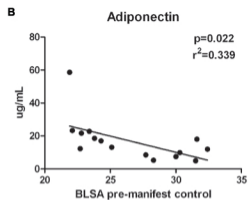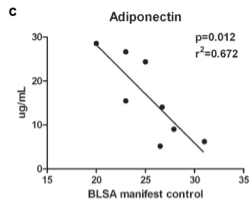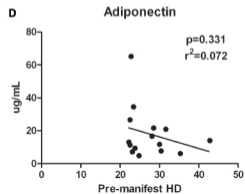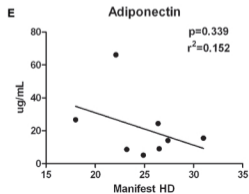

Supplement: Figure S2 — Correlation between adiponectin levels and the body mass index (BMI). Linear regression test was performed using adiponectin levels against the BMI. p ≤ 0.05 was considered statistically significant with respect to the linear correlations' deviation from a zero slope. r2 correlation was calculated by GraphPad Prism v5.0. In familial, BLSA pre-manifest, and manifest control groups, adiponectin levels were negatively correlated with the BMI (A–C); whereas in pre-manifest HD and manifest HD groups no such correlation was observed (D,E). [file Presentation2.PDF]
